# Membrane estrogen signaling in female reproduction and motivation

**DOI:** 10.3389/fendo.2022.1009379

**Published:** 2022-09-29

**Authors:** Caroline S. Johnson, Paul E Micevych, Paul G. Mermelstein

**Affiliations:** ^1^Department of Neuroscience, University of Minnesota, Minneapolis, MN, United States; ^2^Laboratory of Neuroendocrinology, Department of Neurobiology, David Geffen School of Medicine at University of California, Los Angeles, Los Angeles, CA, United States

**Keywords:** estrogen, estrogen receptors, membrane estrogen receptors, metabotropic glutamate (mGlu) receptors, estrogen receptor signaling

## Abstract

Estrogen receptors were initially identified in the uterus, and later throughout the brain and body as intracellular, ligand-regulated transcription factors that affect genomic change upon ligand binding. However, rapid estrogen receptor signaling initiated outside of the nucleus was also known to occur *via* mechanisms that were less clear. Recent studies indicate that these traditional receptors, estrogen receptor-α and estrogen receptor-β, can also be trafficked to act at the surface membrane. Signaling cascades from these membrane-bound estrogen receptors (mERs) not only rapidly effect cellular excitability, but can and do ultimately affect gene expression, as seen through the phosphorylation of CREB. A principal mechanism of neuronal mER action is through glutamate-independent transactivation of metabotropic glutamate receptors (mGluRs), which elicits multiple signaling outcomes. The interaction of mERs with mGluRs has been shown to be important in many diverse functions in females, including, but not limited to, reproduction and motivation. Here we review membrane-initiated estrogen receptor signaling in females, with a focus on the interactions between these mERs and mGluRs.

## Introduction

The estrogen receptors, estrogen receptor-α (ERα) and estrogen receptor-β (ERβ) were initially identified as intracellular, ligand-regulated transcription factors (1), members of the larger nuclear receptor superfamily (2, 3). Originally identified in the uterus (4, 5), these estrogen receptors are expressed throughout the body, including in a multitude of brain regions (6, 7). Estradiol binding to these receptors was initially demonstrated to induce transcriptional changes at estrogen response elements (EREs) (8). However, this classical signaling pathway is not the only mechanism through which estrogen receptors directly elicit genomic change. Many estrogen-regulated genes lack ERE sequences (9, 10), which led to the discovery of additional genomic actions occurring *via* other response elements and transcription factors (11, 12). However, even with multiple pathways leading to direct genomic effects, this was still insufficient to fully explain the plethora of actions estradiol was observed to induce both inside and outside the nervous system.

## Membrane-initiated signaling

The first clues that estrogen signaling could be initiated outside the nucleus came from Szego & Davis in the late 1960s. Following ovariectomy (ovx) in rats, acute exogenous estradiol treatment resulted in an increase in uterine cAMP accumulation within seconds, concentrations indistinguishable from intact animals (13). The speed at which these changes occurred eliminated the possibility of nuclear-initiated action and strongly suggested the recruitment of a surface-initiated second messenger signaling pathway. Rapid effects of estradiol were subsequently noted within the nervous system, first in female preoptic-septal neurons in the hypothalamus. Within seconds of application, estradiol modulated firing rates, returning to experimental baseline when the steroid was removed (14). The use of estradiol conjugated to bovine serum albumen (BSA) further implicated membrane-associated estrogen receptors (15). However, skepticism remained, as there was suspicion that estradiol might be cleaved from BSA (16). Thus, large dendrimer macromolecules conjugated to estrogens were produced. These conjugates avoided the potential for cleaving and were unable to cross the cellular membrane, precluding the activation of nuclear ERs, but still resulted in rapid estradiol signaling (17). While in 2000 a novel estrogen receptor potentially located at the membrane was identified, i.e. G protein-coupled estrogen receptor 1 (GPER1) (18), overexpression of both ERα and ERβ (19), along with immunohistochemical (20) and co-immunoprecipitation studies (21) also indicated that a subpopulation of these classical receptors are trafficked to the membrane (19). The development of transgenic mice allowed researchers to explore the effects of rapid signaling *in vivo*. In transgenic knockout mice devoid of ERα, and/or ERβ, rapid estradiol signaling was eliminated in a brain-region and signaling pathway-dependent manner, suggesting that these receptors are responsible for many of the membrane signaling effects (22).

Membrane-initiated estrogen receptor signaling does not preclude downstream influences on gene expression. Particularly prominent is estradiol activation of PKC-MAPK signaling, ultimately resulting in the phosphorylation of CREB (23–26). Serine-133 phosphorylation of CREB can initiate a diverse array of transcriptional and behavioral changes, including by estradiol-mediated CREB activation *via* membrane ER (mER) interactions with metabotropic glutamate receptors (mGluRs) (23). Initial findings in hippocampal neurons found estradiol acting through ERα-mGluR1a leads to MAPK-dependent CREB phosphorylation. This study elucidated a separate second pathway whereby activation of ERα or ERβ associated with mGluR2 (and Gi/o-mediated inhibition of cAMP) resulted in a decrease in L-type calcium-channel mediated CREB phosphorylation (23). Follow-up studies found mER signaling through mGluR activation throughout the brain, which appears to be a mechanism allowing for diverse signaling outcomes. Not only does mER activation of group I or group II mGluRs activate separate cell signaling pathways, but mER pairing with different group I or II mGluRs (i.e. mGluR1 vs. mGluR5 and mGluR2 vs. mGluR3) can differentially impact neuronal function as well (27).

**Figure 1:**
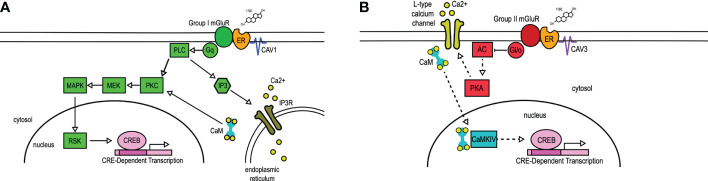
mER transactivation of group I and group II mGluRs. 17β-Estradiol (17βE) binds to membrane-bound estrogen receptors (ER) to activate distinct signaling pathways *via* Group I **(A)** or Group II **(B)** mGluRs. **(A)** Membrane-ER interactions with Group I mGluRs, dependent on CAV1, activates G_q_-mediated signaling through protein lipase C (PLC) and protein kinase C (PKC), subsequent activation of MEK, MAPK, and RSK, and ultimately the phosphorylation of CREB. PLC also activates IP3, which binds to the IP3 receptor (IP3R) to result in the release of intracellular calcium (Ca^2+^). **(B)** Membrane-ER activation of Group II mGluRs, dependent on CAV3, results in the G_i/o_-mediated inhibition of adenylyl cyclase (AC), decreasing the activity (indicated by dashed lines) of protein kinase A (PKA). This results in reduced L-type calcium channel currents and L-type calcium channel-dependent CREB phosphorylation. CaM, Calmodulin; CaMKIV, calmodulin-dependent protein kinase IV; CAV, caveolin; IP3, inositol triphosphate; MEK, MAPK/ERK kinase; MAPK, mitogen-activated protein kinase; RSK, ribosomal S6 kinase. Figure adapted from (32).

The interaction of mERs with mGluRs requires caveolin (CAV) (28–30), a family of scaffolding proteins involved in trafficking receptors to the membrane (31). The particular ER-mGluR pairing is mediated through the CAV isoform associated with the ER (28) (**Figure 1**). A single point mutation in ERα that disrupts receptor localization with CAV1 inhibited estradiol-induced CREB phosphorylation. Reducing CAV1 expression through siRNA knockdown inhibited estradiol-induced CREB phosphorylation while leaving the estradiol-induced L-type calcium channel-dependent decrease in CREB phosphorylation intact. In reciprocal experiments, siRNA knockdown of CAV3 inhibited estradiol-dependent activation of group II mGluRs without affecting estradiol-mediated CREB phosphorylation. In both cases, siRNA knockdown did not grossly impact mGluR signaling, demonstrating the essential nature of caveolin proteins to mER signaling (28). These data contribute to the understanding that CAV1 mediates ERα interactions with group I mGluRs through clustering the receptor at the membrane (28, 29, 33), while CAV3 is involved in the interactions between mERs and group II mGluRs (28). Additionally, an alternatively spliced form of ERα, ERαΔ4, is highly expressed in membrane fractions derived from cultured cells. This receptor has been shown to associate with both mGluR2/3 and CAV3 in ARH membrane fractions (34).

Following these experiments, the precise mechanism of action linking ERs to mGluRs and to the membrane remained unclear, though palmitoylation was an attractive hypothesis. Palmitoylation is a reversible, post-transcriptional modification involved in the trafficking and function of proteins both within and outside the nervous system (35). Global pharmacological blockade of palmitoylation inhibited the downstream outcomes of membrane estradiol signaling, while introducing single point mutations at palmitoylation sites in both ERα and ERβ was sufficient to inhibit membrane signaling (36). Two palmitoyl acetyltransferases (DHHC-7 and DHHC-21) have been shown to be crucial in ER membrane localization (37). Disrupting expression of either was sufficient to inhibit estradiol-dependent CREB phosphorylation (36), and to prevent ERα from associating with CAV1 (38). siRNA knockdown of these enzymes together, but not separately, was sufficient to decrease the palmitoylation of CAV1 itself (38). These data suggest that palmitoylation is a crucial component in the interaction of mERs with CAV proteins, the coupling of mERs with mGluRs, and the subsequent signaling cascades.

While estrogen-mediated signaling plays a crucial role in the female brain, estrogen-mediated signaling is not absent in the male brain. Estrogen plays an important role in masculinizing the brain (39), and rapid estradiol signaling occurs in adult males, including through mGluRs. Estradiol activation of mGluR1a through ERβ modulates sexual behavior in male quails (40, 41), and rodent studies have confirmed rapid mER-mGluR signaling in both the male and female adult cerebellum (42). In females, though, rapid signaling of estradiol, including through mGluRs, has been shown to be incredibly important in driving reproduction, including in the development of the luteinizing hormone surge which stimulates ovulation, the central event in female reproduction. In rodents, and certain other species, rapid membrane signaling is also crucial in the physical display of the principal reproductive behavior, lordosis. Finally, rapid membrane signaling has been shown to play an important role in female motivation for reproduction.

## Ovulation and the luteinizing hormone surge

Ovulation is the central event in female reproduction, controlled by a network of neurons and astrocytes in the hypothalamus that act as a pattern generator, releasing gonadotropin-releasing hormone (GnRH) onto luteinizing hormone (LH) neurons in the anterior pituitary in small, rhythmic pulses (43, 44). Rising estradiol concentrations *via* ovarian release, trigger a switch from an estrogen-negative to an estrogen-positive feedback loop. This estrogen-positive feedback loop, which is unique to females, is crucial in the surge release of LH that ultimately triggers ovulation (45). The preovulatory rise in circulating estradiol sharply increases GnRH neuronal activity and the release of LH from the pituitary to elicit ovulation (46, 47). Blocking either progesterone receptors or progesterone synthesis prevents the surge release of both GnRH and LH and halts the estrous cycle (48, 49). While GnRH neurons do not express ERα or nuclear progesterone receptors (45, 50, 51), kisspeptin neurons that are upstream regulators of GnRH signaling do express the necessary steroid receptors (52–54).

Classically it has been understood that both estradiol and progesterone released from the ovaries orchestrate the LH surge, but it has become apparent that progesterone is also synthesized *de novo* in the brain (55–57), and that it is this neuroprogesterone (neuroP) that is vital in the LH surge that ultimately leads to ovulation (58). Neuroprogesterone is synthesized in hypothalamic astrocytes that express mERα and mGluRs, and it has been shown that the LH surge relies upon the mER-mGluR signaling in these astrocytes (55, 59). Estradiol activation of mERα directly leads to the activation of mGluR1 and its downstream signaling cascades. mGluR1 activity increases inositol triphosphate and allows for the release of intracellular calcium ([Ca^2+^]_i_) stores (59, 60). The release of [Ca^2+^]_i_ activates a Ca^2+^-sensitive adenylyl cyclase (AC-1), which increases the production of cAMP. This cAMP activates protein kinase A (60), leading to the synthesis of neuroP (56, 57, 59). Blocking neuroP synthesis in rats that had both ovaries and adrenals removed is sufficient to prevent the LH surge (61). Cell culture experiments in astrocytes isolated this signaling pathway. Blocking mGluR1a activity, or any part of the cell signaling cascade initiated by the ERα activation of mGluR1, in astrocytes inhibits neuroP synthesis (49, 55, 59, 60). Further, in the absence of estradiol, activating mGluR1a directly is sufficient to release [Ca^2+^]_i_ and induce neuroP synthesis (59, 62).

## Lordosis

Another important aspect of reproduction controlled by ER interactions with mGluRs is lordosis. Lordosis is a reflexive behavior that is acutely triggered by mounting from a conspecific male. This behavior consists of an arching of the spine, the raising of both the head and the hindquarters, and the lifting of the tail (63). While integration of the tactile cues with other externosensory cues is crucial for the display of lordosis, this behavior depends heavily on the appropriate timing of the release of ovarian hormones and the subsequent priming of neural circuits by these hormones. The role of intracellular and membrane-bound ERs, as well as the interaction between mERs and mGluRs, have all been shown to be important components in driving lordosis (64, 65).

**Figure 2:**
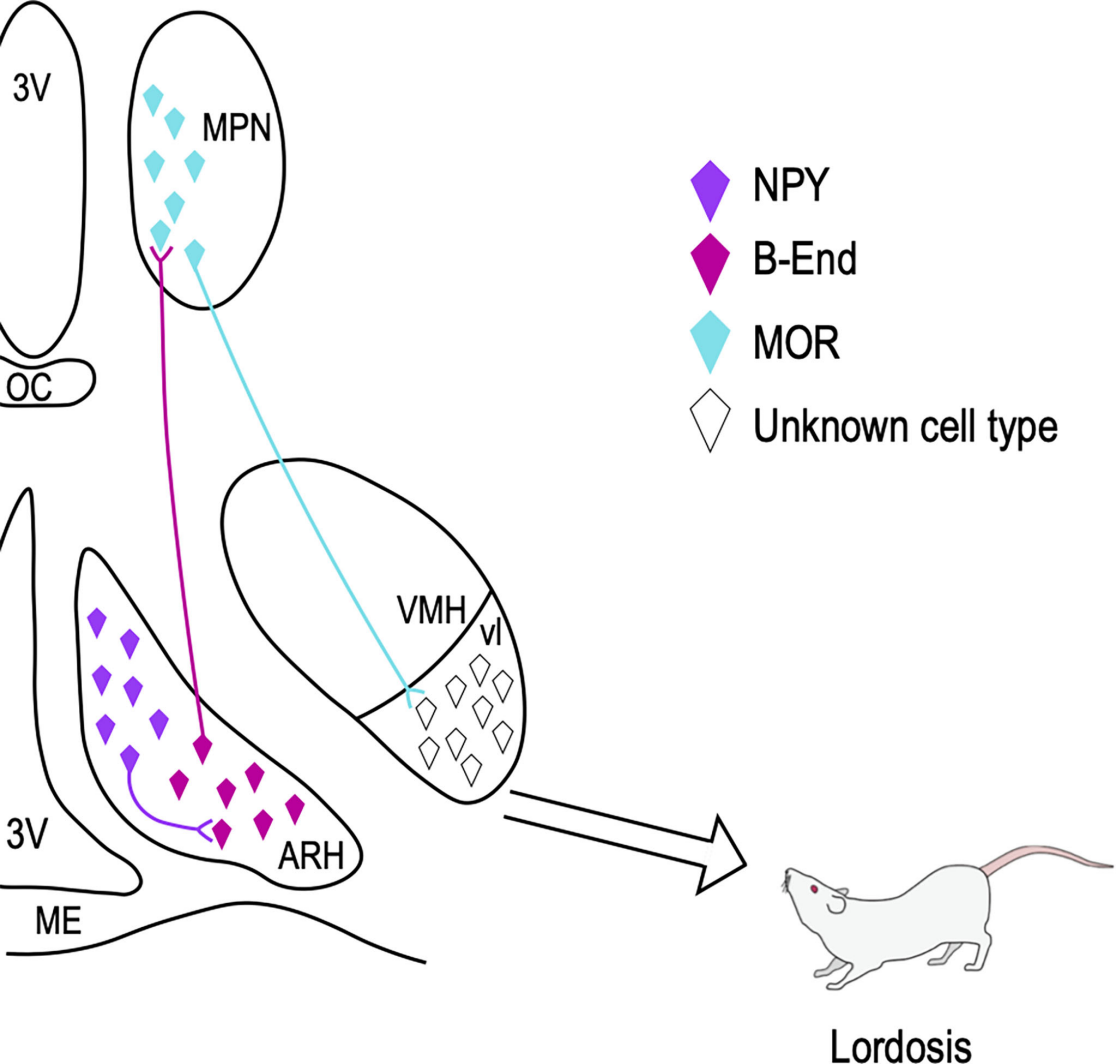
Hypothalamic lordosis circuit. Estradiol acts on estrogen receptor-containing NPY neurons in the ARH, which further project to and activate ARH POMC/B-End neurons. These POMC neurons project to the MPN where the release of B-End activates and internalizes MORs. When these receptors are internalized, lordosis is attenuated. In the ARH, the interaction of mERα & mGluR1a is important for both the internalization of MOR and ultimately the display of lordosis. These MOR-containing neurons in the MPN project further to the ventrolateral (vl) part of the VMH, where signals from other circuits are integrated. Projections from the VMHvl reach lower brain regions which ultimately innervate the spinal motor neurons responsible for the production of the behavior. 3V, 3rd ventricle; OC, optic chiasm; ME, median eminence. Figure adapted from (66).

A core circuit controlling lordosis is within the hypothalamus. Here, signaling between the arcuate nucleus (ARH), the medial preoptic nucleus (MPN), and the ventromedial nucleus of the hypothalamus (VMH) have been shown to be fundamental in the expression of lordosis (64, 66–70) (**Figure 2**). Within this circuit, estradiol first acts on ERα-containing neuropeptide Y (NPY) neurons in the ARH (21, 68, 71), allowing for the release of NPY onto NPY-Y1 receptors in ARH proopiomelanocortin (POMC) neurons. The subset of POMC neurons that are involved in reproduction project further to the MPN where they release β-End onto neurons that express μ-opioid receptors (MORs). The estradiol-induced activation, and subsequent internalization, of MOR depends upon ERα activity (65). Throughout the estrous cycle the activation/internalization of this receptor is out of phase with the ability to express lordosis (69). That is, when MOR is internalized, a measure of activation, the display of lordosis is prohibited. As the cycle progresses, increasing progesterone levels ultimately result in the restoration of MOR to the membrane, a measure indicating that the receptors are not stimulated (72), and the behavior can be expressed. While counterintuitive, this estradiol inhibition of lordosis is necessary for its later full expression. While many neural changes must occur to result in the production of lordosis, recent work has shown that much of the machinery involved in this behavior utilizes fast-acting mER signaling cascades, and particularly those signaling through mGluRs.

Within the ARH, a subset of the NPY neurons express both ERα and mGluR1a, which have been shown to interact at the membrane to initiate signaling (21). The mER-mGluR signaling in the ARH has been shown to be crucial in both the internalization of MOR and the subsequent display of lordosis. The level of estradiol determines the expression of the mERα-mGluR1 complex in the ARH. When estradiol is low, the mERα-mGluR1 complex is present, but as estradiol levels rise the expression of mERα-mGluR1 is reduced (73). In the ARH, antagonizing mGluR1a activity before estradiol treatment is sufficient to attenuate the internalization of MOR in the MPN as well as the ensuing expression of lordosis (20, 21). Activating mGluR1a in the ARH to circumvent the necessity of estradiol is sufficient to result in the internalization of MOR and lordosis (20). Both *in vitro* and *in vivo* activating mGluR1a through estradiol-induced mERα activity increases many important phosphoproteins, including PKC and CREB (20, 21, 23), and the internalization of MOR appears to depend at least in part upon PKC signaling. Downstream from mGluR1a activity, activating PKC signaling in the ARH in the absence of estradiol was sufficient to result in the internalization of MOR, and the amount of this internalization was comparable to that seen following estradiol treatment alone (21).

Another key component in the production of lordosis regulated by fast mER-mGluR activity is morphological changes to neuronal structure. Estradiol affects both the generation and pruning of dendritic spines, though this is not unique to the hypothalamic lordosis circuit but occurs throughout the brain (64, 74, 75) and appears due to retrograde signaling by endogenous opioids (76–78). Within this circuit, important morphological changes can be induced rapidly through mERα-mGluR1a signaling in the ARH. Within 4 hours, estradiol activation of mGluR1a results in an increase in the total number of dendritic spines, which remains for at least 48 hours. By 20 hours these spines display mushroom-shaped morphology, suggesting that these synapses are functional. Blocking mGluR1a activity prevented this spinogenesis, as well as attenuated the display of lordosis (64). Importantly, this time course of changes in morphology lines up with that of the display of lordosis.

## Motivation

Estrogen membrane-receptor signaling has also been found to play a role in motivation. Though the long-term consequence of reproduction is the survival of the species through the production of offspring, the short-term motivation of reproduction is often the immediate drive for the rewarding aspects of the behavior, in females as much as in males (79). While work has focused on the physiology of ovulation and the rodent’s reflexive response to mounting by a male, female sexual behavior is indicative of a motivational drive. Female rats placed in a modified operant chamber, in which the female can choose if and when she wants to copulate, will seek the male for copulation timed to maximize reward (80, 81). Additionally, other pre-copulatory behaviors from female rodents, such as hopping or darting (82, 83), further indicate a level of control over the mating process. This pre-copulatory activity, which is called “pacing,” contributes to a robust dopamine response in the female nucleus accumbens (NAc) in response to mating (84–87). This dopamine response, as well as further structural changes, in the NAc is regulated at least in part by estradiol signaling at the membrane.

The NAc is a key region in reward and incentive salience, and the limbic control of behavioral motivation, and inputs here affect structural morphology and subsequently behavioral output. The limbic system is important in the motivation to engage in reproductive behaviors in both males and females (67, 79), and projections from the hypothalamic nuclei robustly innervate this circuit. The reproductive limbic circuit consists of the MPN, the ventral tegmental area (VTA), and the NAc. A key node connecting the hypothalamus to the limbic component includes the MPN (88). Projections from here reciprocally innervate the mesolimbic dopamine system, including the VTA (89). The VTA projections to the NAc arise from cells that contain ERs (90) and are sensitive to fluctuations in estradiol levels (91), as well as estradiol-mediated signals arising from the MPN (90). These estradiol-mediated changes in VTA signaling have been shown to further affect the subsequent release of DA in the NAc (90).

The predominant output neuron in the NAc is the medium spiny neuron (MSN) - named due to the density of spines it possesses (92). The MSNs in the NAc receive both DAergic and glutamatergic inputs (93), and it has been shown that estradiol plays an important role in modulating both inputs (94–97). MSNs contain few nuclear ERs, suggesting that estradiol acts primarily through membrane-bound receptors ERs (98–103). As in the hypothalamus, estradiol modulates spine density in the NAc and the estradiol-induced morphological changes in the MSNs of the NAc are dramatic in terms of functional circuitry and neuronal morphology (104, 105). In female rodents, sexual experience modulates future sexual behavior through estradiol-mediated morphological changes within the limbic circuit (79). While the complete mechanisms of estradiol modulation on motivational circuity have yet to be fully elucidated, it is likely that mER-mGluR signaling plays a role.

The role of membrane estradiol signaling, and particularly the interaction between mERs and mGluRs, in reproductive motivational drive can be further extrapolated from studies investigating when motivational drive becomes maladaptive, such as in drug addiction. In comparison with men, women tend to show heightened vulnerability to developing a drug addiction (106, 107). Additionally, subjective effects of a drug can vary across the menstrual cycle, as has been reported in response to cocaine. When estrogen levels are high, women report the greatest effects of the drug (108). Interest in the interaction between membrane ERs and group I mGluRs has been taken in understanding the influence of estradiol on drug addiction. In ovx rats, estradiol activation of mGluR5 has been shown to facilitate self-administration of cocaine, while inhibiting this signaling through an mGluR5 antagonist before estradiol administration is sufficient to attenuate this intake of the drug (109). Estradiol activation of mGluR5 in MSNs also results in an increase in the phosphorylation of CREB (100), and a decrease in dendritic spine density in both the core region of the NAc (27, 104, 105). Conversely, estradiol activation of mGluR1 can result in an increase in spine density in the shell region of NAc (27). Taken together, the data suggest that mER-mGluR signaling are important in the drive to seek reward generally, as is apparent in drug taking behaviors, and in the reinforcement of reproductive behaviors.

## Discussion

A great deal of progress has been made in understanding the physiology of rapid estradiol signaling, including the relationship between mERs and mGluRs. Rapid estradiol signaling has been found throughout the brain and the body. In the central nervous system, the signaling cascades initiated by the mER/mGluR complex has been shown to be involved in many physiological functions in both sexes, but particularly in females. In females, the diverse signaling cascades initiated by the interaction of mERs with mGluRs have been shown to play important roles in mediating key aspects of reproduction and motivation, among other crucial functions. While uncovering the roles of CAV and palmitoylation has led to further understanding of this complex signaling cascade, current and future research will inevitably expand our knowledge of mER/mGluR signaling and its physiological and behavioral outcomes.

## Author contributions

CJ wrote the first draft of the manuscript, and revised subsequent drafts. PMe, PMi contributed to and revised the manuscript. All authors read and approved the submitted version.

## Funding

This work was supported by DA041808 and HD10007 to PGM and HD098284 to PEM. CSJ was supported by DA007234.

## Conflict of interest

The authors declare that the research was conducted in the absence of any commercial or financial relationships that could be construed as a potential conflict of interest.

## Publisher’s note

All claims expressed in this article are solely those of the authors and do not necessarily represent those of their affiliated organizations, or those of the publisher, the editors and the reviewers. Any product that may be evaluated in this article, or claim that may be made by its manufacturer, is not guaranteed or endorsed by the publisher.
